# Kinetic analysis of dynamic ^18^F-fluoromisonidazole PET correlates with radiation treatment outcome in head-and-neck cancer

**DOI:** 10.1186/1471-2407-5-152

**Published:** 2005-12-01

**Authors:** Daniela Thorwarth, Susanne-Martina Eschmann, Jutta Scheiderbauer, Frank Paulsen, Markus Alber

**Affiliations:** 1 Section for Biomedical Physics, Clinic for Radiation Oncology, University Hospital Tübingen, Germany; 2 Department of Nuclear Medicine, Radiological Clinic, University Hospital Tübingen, Germany; 3 Department of Radiation Therapy, Clinic for Radiation Oncology, University Hospital Tübingen, Germany

## Abstract

**Background:**

Hypoxia compromises local control in patients with head-and-neck cancer (HNC). In order to determine the value of [^18^F]-fluoromisonidazole (Fmiso) with regard to tumor hypoxia, a patient study with dynamic Fmiso PET was performed. For a better understanding of tracer uptake and distribution, a kinetic model was developed to analyze dynamic Fmiso PET data.

**Methods:**

For 15 HNC patients, dynamic Fmiso PET examinations were performed prior to radiotherapy (RT) treatment. The data was analyzed using a two compartment model, which allows the determination of characteristic hypoxia and perfusion values. For different parameters, such as patient age, tumor size and standardized uptake value, the correlation to treatment outcome was tested using the Wilcoxon-Mann-Whitney *U*-test. Statistical tests were also performed for hypoxia and perfusion parameters determined by the kinetic model and for two different metrics based on these parameters.

**Results:**

The kinetic Fmiso analysis extracts local hypoxia and perfusion characteristics of a tumor tissue. These parameters are independent quantities. In this study, different types of characteristic hypoxia-perfusion patterns in tumors could be identified.

The clinical verification of the results, obtained on the basis of the kinetic analysis, showed a high correlation of hypoxia-perfusion patterns and RT treatment outcome (p = 0.001) for this initial patient group.

**Conclusion:**

The presented study established, that Fmiso PET scans may benefit from dynamic acquisition and analysis by a kinetic model. The pattern of distribution of perfusion and hypoxia in the tissue is correlated to local control in HNC.

## Background

Local control remains a great challenge in head-and-neck cancer (HNC) treatment. Even with an optimal combination of radio- and chemotherapy, local recurrences are observed in up to 50% of the treated patients [1,2]. Up to now, no reliable parameter could be established that would account for this high rate of local failures.

Tumor hypoxia has been known to be associated with poor radiation response for several decades. Recent publications suggested that hypoxia in tumors had a direct influence on treatment success [3,4] by a variety of mechanisms [5,6]. A prognostic impact of tumor hypoxia for therapy outcome in head and neck cancer (HNC) has been shown by different investigators [7-9]. Hypoxia has also been related to lower survival probability and higher risk of recurrence in patients with cervix cancer [4,10]. In these studies, hypoxia was assessed invasively by polarographic Eppendorf electrodes.

Positron emission tomography (PET) with appropriate radiotracers enables non-invasive assessment of the presence and distribution of hypoxia. The radiotracers in frequent use are ^18^F-fluoromisonidazole (Fmiso) [11-13] and chemically similar markers such as ^18^F-fluoroazomycin (Faza) [14] or, with a different binding mechanism, ^60^Cu-ATSM [15]. Some investigations report an unclear correlation between Eppendorf measurements and standardized uptake values (SUV) determined on the basis of Fmiso PET [16]; even though a tumor-to-blood ratio of 1.4 was defined as diagnostic of hypoxia [11]. Thus, the predictive value of Fmiso SUV even several hours after tracer injection remains unclear. Based on their chemical structure, nitroimidazoles are trapped inside hypoxic cells. This feature makes these agents ideal markers for hypoxia in in-vitro cell systems [17]. However, transforming this into larger scale biological systems is problematic and the interpretation of Fmiso PET images remains unclear. An advantage of PET compared to Eppendorf measurements is the ability to display spatial distributions, which is necessary for the integration of hypoxia information into adaptive treatments such as hypoxia dose painting [18-20]. For immunohistochemical investigations, the marker pimonidazole is well established [21-23] to stain hypoxic tumor cells. As the functional binding mechanisms of pimonidazole and Fmiso are similar, Fmiso should be specific to hypoxia to a similar degree. However, the immunohistochemical staining patterns are very complex and reveal a highly heterogeneous distribution of perfused blood vessels and hypoxic patches, sometimes interspersed with necrotic islands, all occurring on a microscopic scale. This may hint as to why Fmiso tracer uptake alone is not a reliable diagnostic quantity, and indicates the requirement of an analysis of dynamic Fmiso PET which takes into account the structural complexity of hypoxic tumor tissues. The study described here was designed to develop a kinetic model in order to understand the spatial and temporal distribution of Fmiso in the tumor tissue. Since the predictive character of Fmiso SUV remains unclear in literature [13,16], the time course of tracer accumulation in the tumor was investigated. This analysis delivers patient specific values for perfusion, kinetic constants and the concentration of tracer retaining cells. Furthermore, the relation between these parameters and radiation therapy (RT) treatment outcome for HNC was investigated in a group of 15 HNC patients who were examined with dynamic Fmiso PET prior to treatment with primary radiotherapy.

## Methods

### Patients

After informed consent, sixteen patients (mean age: 57.2 years old, range: 46 – 69; 14 male, 2 female) with advanced stage head and neck cancer (HNC) were examined between November 2001 and March 2004. The Fmiso examinations were performed prior to radiation therapy (RT) treatment. All patients were treated with primary RT to 70 Gy. Three of these patients were treated with Intensity Modulated Radiotherapy (IMRT) in 35 fractions, 5 fractions a week with a daily dose of 2 Gy. The other 13 patients received conventional RT, 5 fractions with 2 Gy per week until 30 Gy. This first phase was followed by a hyperfractionation composed of a dose of 1.4 Gy applicated twice per day until the end of treatment. In addition, concomitant chemotherapy was prescribed for 14 patients. Seven patients received 5-Fluorouracil/Mitomycin chemotherapy, whereas for six patients Cisplatin/Mitomycin was prescribed; one patient had Paclitaxel/Cisplatin chemotherapy. Whenever possible (*n *= 12), an additional [^18^F]-fluorodeoxyglucose (FDG) PET was taken a few days (1 – 3) before or after the Fmiso PET scan. For each patient, additional computed tomography (CT) image data was available. These CT scans, on which delineation of target volumes and organs at risk was performed, were used for RT treatment planning.

After the end of therapy, patients were reviewed regularly every three months with clinical examination, flexible endoscopy and computed tomography (CT) when recurrent disease was suspected. Routine CT scans were also acquired six weeks and one year after therapy was finished. Failure was defined as CT proven tumor progression.

### Data acquisition

The Fmiso PET examinations were performed on a whole-body scanner (Advance, GE Medical Systems, Milwaukee, US) after automatic bolus injection of 400 MBq Fmiso. PET data acquisition was started at the time of tracer injection. During the first 15 (9 patients) to 60 min (7 patients), a dynamic image acquisition of 31 (40) frames was performed. Additional static emission scans were taken 2 h and 4 hour post injection (p.i.). Concerning the FDG PET acquisition, a static emission scan was taken 1 h after injection of approximately 400 MBq FDG.

For the delineation of the tumor volume relevant in the context of this study, the FDG PET image data was used. The tumor volume was defined as the volume including all voxels with at least 40% of the maximum intensity. This delineation technique was combined with a 12 mm margin (3 PET voxels). The tumor volume variable *V *used in the current study refers to the described FDG PET volume. It is determined as *V *= *n·v*, where *n *is the number of tumor voxels. *v *represents the volume of a single voxel, in our case *v *= (0.4^2^·0.425) cm^3 ^= 0.068 cm^3^. In order to match the FDG-defined tumor volume onto the three different Fmiso data sets (dynamic, 2 and 4 h p.i.), an automatic coregistration [24] was performed, which achieved a matching accuracy of ≤ 2 mm. The resulting transformation matrices were used to determine a time-activity curve (TAC) for each tumor voxel.

### Compartment model

**Figure 1:**
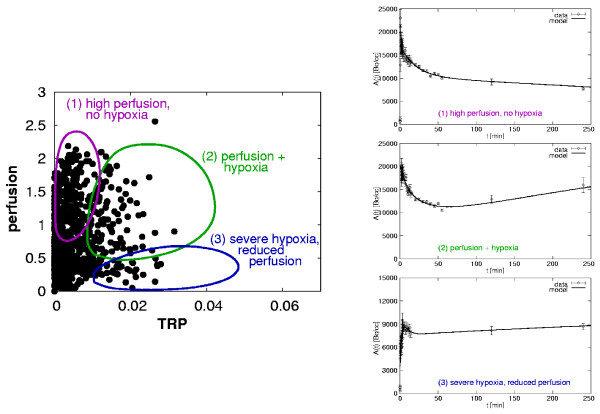
*Left*: Scatter plot for one patient based on tracer retention and perfusion parameters resulting from a kinetic Fmiso analysis. Schematically shown are typical regions for characteristic perfusion-hypoxia patterns: (1) High perfusion without hypoxia, (2) well perfused and simultaneously hypoxic, and (3) severe hypoxia, low vessel density. *Right*: Corresponding types of characteristic Fmiso Time-Activity Curves.

**Figure 2:**
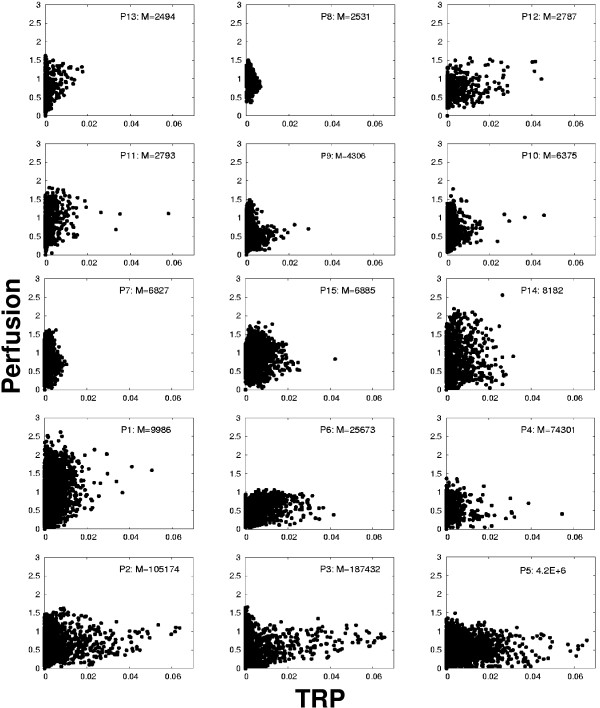
Scatter plots of all 15 patients with increasing *M*-value.

The voxel-by-voxel TACs were analyzed using a pharmaco-kinetic model which is described in detail elsewhere [25]. Briefly, the kinetic model consists of two compartments, one corresponding to the irreversible binding of the tracer in hypoxic cells, the other representing freely diffusive Fmiso. This two-compartment system is combined with an input function which is individually determined by a reference tissue approach for lack of a blood signal in the field of view of the scanner (see [25] for details). The voxel-by-voxel analysis of the Fmiso TACs was done by fitting the five-parameter analytical model function for the tracer concentration in the tissue compartments to the measured Fmiso curves. This approach yields for each tumor voxel one characteristic value for tracer retention and perfusion. The perfusion value, i.e. the density of perfused blood vessels in the respective tumor voxel, is mainly guided by the shape of the TAC during the first few minutes after injection. In contrast, the amount of tracer retention potential (TRP) in the voxel is related to the properties of the curve several hours after tracer injection. TRP takes into account the number of viable hypoxic cells as well as their grade of hypoxia. In other words, TRP is a measure of the concentration of specifically bound tracer in the considered area. Fluctuations in perfusion states are taken into account by construction of the model [25]. In order to visualize TRP and perfusion characteristics of the whole tumor simultaneously, a *scatter plot *is introduced (see appendix and fig. 1). In this plot, the TRP in a voxel is plotted along the *x*-axis, while the contribution to the signal from perfused blood vessels is plotted along the *y*-axis. The variety of scatter patterns in the patient group leads to the hypothesis that TRP and perfusion are independent and spatially variable parameters of a tumor tissue (see figure 2).

### Data analysis and statistics

Tumor control was defined on the basis of computed tomography (CT) scans as complete and persistent regression of the primary tumor and failure was defined as local recurrence of the tumor in the irradiated fields. Follow up time was determined from the end of RT treatment until the day of the last CT. Different variables that might influence treatment outcome were compared using the Wilcoxon-Mann-Whitney (Wilcoxon signed rank) *U*-test between patient groups showing no local relapse and failure. In all cases, a two-sided significance level of 0.05 was used. Correlation of different variables with was assessed using a Pearson correlation coefficient.

The impact on treatment outcome was checked for different classes of variables: tumor volume and patient age, SUV related factors and variables derived from the kinetic analysis. The SUV related factors were the maximum standardized uptake value (SUV_*max*_) and the fractional hypoxic volume (FHV) 4 h after Fmiso injection. FHV is defined as the fraction of tumor volume presenting a tumor-to-blood ratio larger than 1.4. Both variables SUV_*max *_and FHV have been correlated with tumor hypoxia in earlier studies [11,13]. Finally, a number of parameters derived from the compartmental analysis were checked for a statistically significant influence on therapy outcome. These parameters were the mean value of TRP, the mean value of perfusion, and two metrics involving both TRP and perfusion parameter values. A first metric was defined intuitively as the volume integral of the TRP-to-perfusion ratio (HPR). A second metric, which was derived from a model of tumor dose-response and reoxygenation, is the *malignancy value M *as described in more detail in the appendix.

## Results

### Kinetic Fmiso data

The voxel-by-voxel Fmiso TACs showed a variety of different tracer uptake patterns. Perfusion and hypoxia status of the tissue area can be differentiated by means of the Fmiso TAC shape. The former is determined by the part of the TAC corresponding to time points only a few minutes p.i, whereas the latter is linked to the shape of the curve several hours after tracer injection. The TACs observed for the group of 16 patients showed mainly three different types of perfusion-hypoxia patterns which correspond to (1) tissue areas with a high vessel density, (2) well perfused but also hypoxic, and (3) severely hypoxic tumor areas (see fig. 1).

### Model

The presented compartment model allows us to derive patient specific perfusion-hypoxia patterns. The model is able to describe the different observed types of Fmiso time curves. Characteristic TACs are associated to distinct areas in the scatter plot (figure 1), which indicates high stability of the model. The patterns for the whole group of patients are displayed in scatter plots in figure 2 (appendix). The ultimate purpose of the kinetic model is to subtract the background of unbound tracer from the signal intensity.

### Patients

Characteristics of the group of 15 patients are summarized in table 1. For the examined patient group, the follow up time was in the range of 2 – 21 months (median: 12.8 months). Patients were 46 to 68 years old (median: 59 years). FDG-tumor volumes ranged from 32.4 to 287.6 cm^3 ^with a median volume of 114.5 cm^3^. Overall, 7 of the 15 patients had local recurrences. All observed failures occurred in the first 8 months after the end of therapy.

**Table 1:** patient characteristics. Tumor characteristics of the examined patients.

Patients						
Pat. nr.	primary tumor site	age	sex	TNM stage	tumor volume *V *[cm^3^]	failure site

1	oropharynx	60	m	T4 N2b MO	258.1	T*/N^†^
2	oropharynx	51	m	T4 N2c MO	126.2	T*
3	larynx	66	m	T4 N2c MO	153.7	T*/N^†^
4	FOM^‡^	46	m	T3 N2b MO	59.0	T*
5	BOT^§^	49	m	T4 N2c MO	287.6	T*/N^†^
6	oropharynx	48	m	T2 N2c MO	114.7	T*/N^†^
7	FOM^‡^	68	m	T4 Nl M0	213.7	T*/N^†^
8	oropharynx	65	m	T3 N2c MO	74.3	-
9	hypopharynx/BOT^§^	51	m	T4 N2c MO	100.9	-
10	oropharynx	59	m	T2 N3 MO	172.4	-
11	oropharynx	50	f	T4 N2c MO	44.0	-
12	larynx-/hypopharynx	60	m	T4 NO MO	32.4	-
13	oro-/hypopharynx	49	m	T3 N2c MO	80.9	-
14	oropharynx	60	f	T4 N2b MO	52.4	-
15	unknown	68	m	T4 Nl M0	125.5	-

*T: tumor; ^†^N: node.

^‡^FOM: floor of mouth; ^§^BOT: base of tongue.

Image analysis of the Fmiso PET scans taken 4 h p.i. revealed maximum SUVs in the tumor volume between 1.36 and 4.02. The median SUV_*max *_was 2.25. The FHV ranged from 0 to 72.5% with a mean of 19.7%. Due to the chosen tumor volume definition strategy, which implies the addition of a margin, the determined FHV can never reach 100%.

Examination of the scatter plots showed very different patterns of hypoxia and perfusion. All possible combinations of hypoxia and perfusion parameters were observed: well perfused tumors which were not at all hypoxic, tumors showing at the same time a quite high vascular density and hypoxic subareas, and finally also tumors that were badly perfused and severely hypoxic. These two variables represent physiological tumor characteristics that are not correlated (*r *= -0.096). As a first result, it has to be stated that hypoxia occurs independently from the degree of perfusion in tumor tissues.

The Wilcoxon-Mann-Whitney *U*-test with respect to the age of the patients showed no difference (*P *= 0.3) between the subgroups with and without relapse. In contrast, there was a significant difference in tumor volume between the two subgroups (*P *= 0.014). This corroborates the findings of earlier studies that correlated tumor size with treatment outcome [26]. Also, SUV_*max*_, determined 4 h after injection separated patients according to failure and progression free survival (PFS). The significance for SUV_*max *_was only weak *P *= 0.041, whereas the *U*-test for the FHV showed no significance at all (*P *= 0.13).

Regarding the variables derived from the kinetic analysis, mean tumor perfusion and HPR discriminated between the group without recurrence and the failure group (*P *= 0.05 and 0.008, respectively). The mean TRP value showed no significance (*P *= 0.18). Finally, the malignancy value *M *was highly significant, with *P *= 0.0013 (table 2). The prognostic value of this model based metric *M *is higher than the value of tumor size or SUV_*max *_after 4 h.

**Table 2:** results of statistical analysis. Results of univariate analysis of prognostic factors.

Results *U*-Test	
variables	*P*-Value

age [years]	NS* (0.30)
tumor volume *V *[cm^3^]	0.014
SUVmax† MathType@MTEF@5@5@+=feaafiart1ev1aaatCvAUfKttLearuWrP9MDH5MBPbIqV92AaeXatLxBI9gBaebbnrfifHhDYfgasaacH8akY=wiFfYdH8Gipec8Eeeu0xXdbba9frFj0=OqFfea0dXdd9vqai=hGuQ8kuc9pgc9s8qqaq=dirpe0xb9q8qiLsFr0=vr0=vr0dc8meaabaqaciGacaGaaeqabaqabeGadaaakeaacqqGtbWucqqGvbqvcqqGwbGvdaqhaaWcbaWexLMBbXgBcf2CPn2qVrwzqf2zLnharyGvLjhzH5wyaGabciaa=1gacaWFHbGaa8hEaaqaaiabcccigcaaaaa@3F10@	0.041
FHV^‡ ^[%]	NS* (0.13)
mean TRP*	NS* (0.18)
mean perfusion^§^	0.05
HPR^§^	0.008
malignancy value *M*^§^	0.001

*NS: not significant (*p *> 0.05); ^†^SUV_*max*_: maximum SUV; ^‡^FHV: fractional hypoxic volume.

^§^derived from kinetic model

## Discussion

Recent publications revealed contradictory results concerning the correlation of static Fmiso PET data and tumor hypoxia [11-13,16]. As the irregular architecture of tumors complicates Fmiso uptake, a kinetic model was developed in order to analyze spatial and temporal distribution of the tracer in head-and-neck tumors. The presented model enables to differentiate between tumor perfusion and hypoxia. This feature of the model constitutes the link between Fmiso distribution and retention and the structural architecture of the tumor tissue.

The results of this study showed, that SUV_*max *_alone even at late time points has limited predictive value. These findings are in line with results of other investigators [16] who found that SUV 2 h p.i. and Eppendorf did not correlate well.

A limiting factor for the retention of Fmiso in the tumor is that binding of the tracer can only take place in *viable *hypoxic cells which may be few if the tumor is largely necrotic. In other words, a low level of the Fmiso TAC several hours after tracer injection is not necessarily due to non-hypoxic tissue. This might also be caused by largely necrotic tumor areas which contain only a very low number of strongly hypoxic cells. In this case, the low intensity of the PET signal would lead to an underestimation of the extent of hypoxia by the SUV-method. A kinetic analysis subtracts the non-specific background signal and hence enables to determine the local TRP of the tumor. Still, the classical hypoxic tumor core may only give a weak signal due to the low density of tracer retaining cells. Hence, a second parameter is needed to give a more complete picture of the abnormalities of the tissue architecture.

The analysis of the parameters derived from the kinetic model demonstrated, that TRP and perfusion values alone do not predict treatment outcome. Additionally, hypoxia occurred independent of degree of perfusion, since no correlation was found between the two variables. Recent immunohistochemical investigations of simultaneous pimonidazole and blood vessel staining of tissue sections [21-23] revealed the co-existence of hypoxic areas and perfused blood vessels. These results were corroborated in our study. Taking both parameters together proved to be reliable predictors for treatment outcome. The malignancy metric *M*, which involves these two physiological characteristics of the tissue, was found to be the strongest prognostic factor.

Most essential for the design of new adaptive treatment strategies is the time until reoxygenation takes place after the beginning of RT. The malignancy metric *M *involves an estimate of this characteristic time. The worst physiological setting in a tumor seems to be the combination of low perfusion and severe hypoxia, as reoxygenation then appears to be very slow. In contrast, a high degree of perfusion co-existing with hypoxic areas may favor fast reoxygenation. Hence, this setting might be associated with an intermediate level of risk. This interpretation can be supported by follow-up scans during RT, which will be reported in a future publication.

Fmiso uptake kinetics are quite slow due to long diffusion distances and for lack of active transport mechanisms. PET scans several hours after injection of the radiotracer are therefore essential. Nevertheless, dynamic scans at short times p.i. cannot be abandoned, as they are needed to determine the degree of perfusion of the tumor.

There is no possibility in Fmiso PET to distinguish between acute and chronic hypoxia [27]. On one hand, this is due to a quite large size of the image voxels (≈ (4 mm)^3^). On the other hand, the slow kinetics of tracer retention do not allow a distinction of fast re-perfusion. Since both effects are a consequence of the deficient vasculature, they may co-exist anyway.

The results of this study demonstrate that Fmiso PET has prognostic value for therapy outcome, but only when perfusion and retention are both taken into consideration. A higher predictive value was associated to the malignancy value *M *derived form kinetic analysis than to tumor volume or SUV based variables. Hence, Fmiso PET might in the future be used to individually select patients for an adapted radiotherapy treatment as e.g. dose painting [18-20]. Furthermore, variables derived from a kinetic analysis [25] may serve to determine individual dose escalation factors in order to overcome hypoxia related treatment resistance.

## Conclusion

The interpretation of Fmiso PET examinations with respect to hypoxia benefits greatly from a kinetic analysis. The presented kinetic analysis determines hypoxia and perfusion parameters, which were shown to be able to stratify patient groups according to RT treatment outcome. The results of this explorating, hypothesis generating study require validation in a larger group of patients.

## Appendix

### Scatter plots

By virtue of the kinetic model analysis of the time-activity curves of tracer uptake, it is possible to eliminate the non-specific background activity in the signal. The model has five fit parameters, which are determined for each voxel of the tumor volume. Two parameters are of special interest: the relative contribution of the perfused blood vessels, short *W*_*P*_, which dominates the signal during the first few minutes after injection. Further, the tracer retention potential *R*, which is a combination of the concentration of retaining cells and the kinetic constants of the reaction. In a scatter plot, the values of *R *are plotted along the *x*-axis, and the values of *W*_*P *_are plotted along the *y*-axis for all voxels of the tumor volume. The scatter plots of all 15 patients in figure 2 show clearly that both values are independent variables and vary widely in a population.

### TCP model

The resistance of a hypoxic tumor to RT is governed, among others, by two factors: the initial magnitude of the hypoxic subpopulation of clonogenic cells, and the rate with which these cells are reoxygenated. We assume that the former is related to *R*, while the latter is related to *W*_*P*_. The rationale for the second assumption is, that in areas where the blood vessel density is high, hypoxic cells have a greater chance to become oxic quickly. Conversely, if the vasculature is severely deficient, reoxygenation is delayed. The common Poisson model of tumor control probability (TCP) states:

−ln⁡TCP (D) = ∑in exp⁡(−α0D)     (1)
 MathType@MTEF@5@5@+=feaafiart1ev1aaatCvAUfKttLearuWrP9MDH5MBPbIqV92AaeXatLxBI9gBaebbnrfifHhDYfgasaacH8akY=wiFfYdH8Gipec8Eeeu0xXdbba9frFj0=OqFfea0dXdd9vqai=hGuQ8kuc9pgc9s8qqaq=dirpe0xb9q8qiLsFr0=vr0=vr0dc8meaabaqaciGacaGaaeqabaqabeGadaaakeaacqGHsislcyGGSbaBcqGGUbGBcqqGubavcqqGdbWqcqqGqbaucqqGGaaicqqGOaakcqWGebarcqqGPaqkcqqGGaaicqqG9aqpcqqGGaaidaaeqbqaaiabd6gaUjaaykW7cyGGLbqzcqGG4baEcqGGWbaCaSqaaiabdMgaPbqab0GaeyyeIuoakiabcIcaOiabgkHiTiabeg7aHnaaBaaaleaacqaIWaamaeqaaOGaemiraqKaeiykaKIaaCzcaiaaxMaadaqadaqaaiabigdaXaGaayjkaiaawMcaaaaa@4EB7@

where the sum runs over all voxels *i *of the tumor and *n *is the number of cells per voxel. *D *is the total dose and *α*_0 _the radiation sensitivity of a non-hypoxic cell. We modify this to include hypoxic subpopulations to read

−ln⁡ TCP(D) = ∑i∫dαh nhi(αh) exp⁡(−(α0−αh)D)     (2)
 MathType@MTEF@5@5@+=feaafiart1ev1aaatCvAUfKttLearuWrP9MDH5MBPbIqV92AaeXatLxBI9gBaebbnrfifHhDYfgasaacH8akY=wiFfYdH8Gipec8Eeeu0xXdbba9frFj0=OqFfea0dXdd9vqai=hGuQ8kuc9pgc9s8qqaq=dirpe0xb9q8qiLsFr0=vr0=vr0dc8meaabaqaciGacaGaaeqabaqabeGadaaakeaacqGHsislcyGGSbaBcqGGUbGBcaaMc8UaeeivaqLaee4qamKaeeiuaaLaeeikaGIaemiraqKaeiykaKIaeeiiaaIaeeypa0JaeeiiaaYaaabuaeaadaWdbaqaaiabdsgaKjabeg7aHnaaBaaaleaacqWGObaAaeqaaaqabeqaniabgUIiYdaaleaacqWGPbqAaeqaniabggHiLdGccaaMc8UaemOBa4MaemiAaG2aaSbaaSqaaiabdMgaPbqabaGccqGGOaakcqaHXoqydaWgaaWcbaGaemiAaGgabeaakiabcMcaPiaaykW7cyGGLbqzcqGG4baEcqGGWbaCcqGGOaakcqGHsislcqGGOaakcqaHXoqydaWgaaWcbaGaeGimaadabeaakiabgkHiTiabeg7aHnaaBaaaleaacqWGObaAaeqaaOGaeiykaKIaemiraqKaeiykaKIaaCzcaiaaxMaadaqadaqaaiabikdaYaGaayjkaiaawMcaaaaa@6505@

= n exp⁡(−α0D) ∑i∫dαh hi(αh) exp⁡(αhD).     (3)
 MathType@MTEF@5@5@+=feaafiart1ev1aaatCvAUfKttLearuWrP9MDH5MBPbIqV92AaeXatLxBI9gBaebbnrfifHhDYfgasaacH8akY=wiFfYdH8Gipec8Eeeu0xXdbba9frFj0=OqFfea0dXdd9vqai=hGuQ8kuc9pgc9s8qqaq=dirpe0xb9q8qiLsFr0=vr0=vr0dc8meaabaqaciGacaGaaeqabaqabeGadaaakeaacqGH9aqpcqqGGaaicqWGUbGBcaaMc8UagiyzauMaeiiEaGNaeiiCaaNaeiikaGIaeyOeI0IaeqySde2aaSbaaSqaaiabicdaWaqabaGccqWGebarcqGGPaqkcaaMc8+aaabuaeaadaWdbaqaaiabdsgaKjabeg7aHnaaBaaaleaacqWGObaAaeqaaOGaaGPaVlabdIgaOnaaBaaaleaacqWGPbqAaeqaaaqabeqaniabgUIiYdGccqGGOaakcqaHXoqydaWgaaWcbaGaemiAaGgabeaakiabcMcaPiaaykW7cyGGLbqzcqGG4baEcqGGWbaCaSqaaiabdMgaPbqab0GaeyyeIuoakiabcIcaOiabeg7aHnaaBaaaleaacqWGObaAaeqaaOGaemiraqKaeiykaKIaeiOla4IaaCzcaiaaxMaadaqadaqaaiabiodaZaGaayjkaiaawMcaaaaa@613E@

Here, *h*(*α*_*h*_) is the frequency with which an average reduction of the cell sensitivity by *α*_*h *_occurs over the course of the treatment. The integral is the malignancy *M*. We define a phenomenological relation between the kinetic model parameters and the malignancy by:

*M *= 1 + exp(*bR*/(*W*_*P *_+ *c*)),     (4)

where *b *and *c *are fit parameters. Finally, we obtain

−ln⁡ TCP(D)=a∑i[1+exp⁡(bRi/(WP,i+c))]     (5)
 MathType@MTEF@5@5@+=feaafiart1ev1aaatCvAUfKttLearuWrP9MDH5MBPbIqV92AaeXatLxBI9gBaebbnrfifHhDYfgasaacH8akY=wiFfYdH8Gipec8Eeeu0xXdbba9frFj0=OqFfea0dXdd9vqai=hGuQ8kuc9pgc9s8qqaq=dirpe0xb9q8qiLsFr0=vr0=vr0dc8meaabaqaciGacaGaaeqabaqabeGadaaakeaacqGHsislcyGGSbaBcqGGUbGBcaaMc8UaeeivaqLaee4qamKaeeiuaaLaeeikaGIaemiraqKaeiykaKIaeyypa0Jaemyyae2aaabuaeaacqGGBbWwcqaIXaqmcqGHRaWkcyGGLbqzcqGG4baEcqGGWbaCaSqaaiabdMgaPbqab0GaeyyeIuoakiabcIcaOiabdkgaIjabdkfasnaaBaaaleaacqWGPbqAaeqaaOGaei4la8IaeiikaGIaem4vaC1aaSbaaSqaaiabdcfaqjabcYcaSiabdMgaPbqabaGccqGHRaWkcqWGJbWycqGGPaqkcqGGPaqkcqGGDbqxcaWLjaGaaCzcamaabmaabaGaeGynaudacaGLOaGaayzkaaaaaa@598C@

with *a *= *n exp*(-*α*_0_*D*) as an additional fit parameter. This sum can be computed as a sum over the points of a scatter plot.

The parameters *a*, *b *and *c *were determined by a maximum log-likelihood fit of TCP to the group of 15 patients in this study. Their values obtained as *a *= 5.7·10^-5^, *b *= 198.6 and *c *= 0.565. The goodness of fit was estimated by evaluation of the deviance Δ. The deviance is defined as twice the difference between the current and the full log-likelihood Δ = -2(*L*_*c *_- *L*_*f*_), which is supposed to follow a *χ*^2 ^distribution. In our case, the deviance confirmed an acceptable fit (Δ = 2.75, *p *> 0.05).

**Figure 3:**
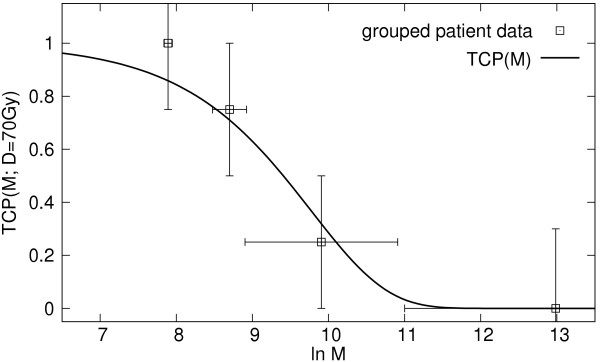
TCP curve fitted to the outcome data of 15 patients irradiated with a total dose of 70 Gy.

Figure 3 shows the the fitted TCP curve as a function of the malignancy value *M *together with the grouped data points obtained from outcome data for the group of 15 patients in this study.

## Competing interests

The author(s) declare that they have no competing interests.

## Authors' contributions

DT developed the kinetic model, the parameter analysis strategy, and the TCP model, performed all data analysis, and drafted the manuscript. SE carried out all PET examinations and acquired the data. FP was involved in the design of the study and its coordination. JS participated in the design of the study and performed the statistical analysis. MA conceived the study, developed the kinetic model and the TCP model, has made substantial contributions for data interpretation and was participated in drafting the manuscript. All authors read and approved the final manuscript.

## Pre-publication history

The pre-publication history for this paper can be accessed here:


